# Evaluation of PathTezt^™^, a Liquid-Based Cytology System

**DOI:** 10.31557/APJCP.2021.22.10.3261

**Published:** 2021-10

**Authors:** Nur Syuhada Mohd Nafis, Anani Aila Mat Zin

**Affiliations:** 1 *Hospital Universiti Sains Malaysia,16150 Kubang Kerian, Kelantan, Malaysia. *; 2 *Department of Pathology, School of Medical Science, Health Campus, Universiti Sains Malaysia, Kubang Kerian, Kelantan, Malaysia. *

**Keywords:** Liquid-based cervical smear, cost-effectiveness, PathTezt processor

## Abstract

Liquid-based preparation (LBP) cytology is commonly used in most laboratories these days due to its convenience and reliable results for the cervical cancer screening program. The PathTezt™ Liquid-based Pap smear is a second-generation LBP, which uses a filter-based concentration technique in processing the sample.

**Objective:**

This study was done to evaluate the cellular fixation, morphology, quality of smear in gynae cytology, and diagnostic interpretation of cervical cytological smears produced by the PathTezt liquid-based processor.

**Materials and Methods:**

A total of 400 pap smear samples were taken and processed using the PathTezt 2000 processor. The slides were evaluated in terms of sample adequacy, percentage of the circle covered by epithelial cells, cellular distribution, obscuring factors, and cell fixation.

**Results:**

About 95.25% (381) of the samples were satisfactory for the evaluation. In 19 (4.75%) of the samples, epithelial cells covered less than 50% of the circle. A sample with good cellular distribution was seen in 92% of the cases, while 354 (88.5%) samples showed minimal inflammatory background. Almost all the smears (95.75%) had no erythrocytes in the background. All smears showed good quality fixation features toward nuclear, cytoplasm, and microorganisms. The total performance rate was 99%.

**Conclusion:**

Although the PathTezt liquid-based processor is still new compared to other first-generation LBP, the smears produced by this method were of high quality and it was cost-effective.

## Introduction

Cervical cancer is the most common malignant cancer of the female reproductive organs worldwide and the third most common cancer among women in Malaysia (Ab Manan et al., 2019). According to the Malaysia Ministry of Health guideline for the early detection of common cancer, women at the age of 20 to 65 years old who have had sexual intercourse should be screened for cervical cancer. Cervical cancer screening is a secondary preventive plan and carried out for early detection of cervical cancer. The screening program will not prevent a person from getting an human papillomavirus (HPV) infection, but it can detect abnormalities that can be treated early. There are two types of screening tests available, namely cervical cytology (the Pap test)and molecular screening (HPV DNA testing). The Pap smear is a well-known and useful tool for detecting pre-cancerous and cancerous cells in the cervix ever since it was invented. Pap smear screening is divided into conventional smears and liquid-based preparation (LBP) smears (Mohd Taib et al., 2020).

As technology evolved, the use of conventional smears has become less relevant, and more laboratories have turned to LBP smears instead. The liquid-based preparation smear promised a good quality slide with a cleaner background, less interference from inflammatory cells, and one flat layer of cells, unlike conventional smears.

Thin Prep^®^ by Hologic Inc. has become a pioneer in liquid-based preparation cytology since it was first invented in 1996, then followed by SurePathTM by Beckton Dickinson in 1999. The United States Food and Drug Administration (FDA) has approved both LBP systems (Bibbo and Wilbur, 2015). In Malaysia, most laboratories use first-generation liquid-based preparations for cytology such as ThinPrep and SurePath. Both LBP systems have a long history as well as the pros and cons for the users. ThinPrep uses a filter-based concentration technique as its principal method, while SurePath uses the cell enrichment method (Desai, 2014).

PathTezt™ System (Biocytech Corporation) can be considered as a second-generation LBP cytology (Biocytech Corporation, 2015). The company was based in Ipoh, Malaysia. They offer the same method as ThinPrep but with additional features, such as processing the sample in the presence of cytobrush (Biocytech Corporation, 2016; Bibbo and Wilbur, 2015; Hologic Inc., 2017). Not much study or comparison has been done to compare and evaluate the Path Tezt system with other LBP systems. As a research university, Hospital Universiti Sains Malaysia conducted a study to test the system to demonstrate whether it can compete with the first-generation LBP given that being more economical for small laboratories in developing countries. This study aimed to evaluate the cellular fixation, morphology, and quality of smears in gynaecology cytology as well as the diagnostic interpretation of cervical cytological smears produced by the PathTezt as a liquid-based cytology procedure.

## Materials and Methods

A total of four hundred (400) PathTezt cervical smear kits were distributed to the Obstetrics and Gynaecology (O and G) Clinic and Klinik Rawatan Keluarga (KRK) at Hospital Universiti Sains Malaysia. All the samples were collected using the PathTezt pap smear kit . PathTeztTM is an automated computer-controlled device designed to do standardized thin-layer cytological cell preparations using a filtration system (Phaliwong, et al., 2018). All samples were taken from the cervix area only, not from the vagina area. Smears from pregnant women or post-hysterectomy patients were excluded from the study.

All the samples were processed using the PathTezt 2000 Processor. Using the filter-based concentration technique, the sample was first vortexed (cell dispersion), then poured onto a filter by vacuum suction. The cell was then transferred to the glass slide before being immediately dropped into an alcohol bath for fixation (Biocytech Corporation, 2016). All smears were stained with Papanicolaou stain using the regressive method. Smears were distributed among certified cytoscreeners for evaluation, and then were verified by cytopathologists for definitive evaluation. The variables of (i) sample adequacy, (ii) percentage of the circle covered by epithelial cells, (iii) cellular distribution, (iv) obscuring factors, and (v) cell fixation were analysed. To obtain sample adequacy and the percentage of the circle covered by epithelial cells, we used “The Bethesda System for Reporting Cervical Cytology 2014” and the diagram made by Dr. Euphemia MgGoogan, a senior lecturer in Pathology from the University of Edinburg, respectively (Desai, 2014).

Cellular distribution was analysed to find out whether the cells were evenly dispersed or overlapped on the smear or not. To investigate obscuring factors, the grade was obtained according to the percentage of red blood cells and inflammatory cells obscuring the smear. For studying cell fixation, it was graded in terms of the quality of nuclear, cytoplasmic, and organism (bacterial, fungi, and protozoa) preservation features. Each of the variables was graded from 1 to 3 (1: poor, 2: average, and 3: good ). A total score below 9 was regarded as poor, 10 to 15 as average, and 16 to 20 as good. The recommendation for the system is based on the percentage of smears with a good score.

*Statistical analysis*

Chi-square test was used to discover any relationships between total score and the criteria for scoring and data agreement in between histological-cytological correlation. The association was determined using multiple logistic regression tests. All calculations were performed by using SPSS version 26. P-value < 0.05 was taken as statistically significant.

## Results

**Figure 1:**
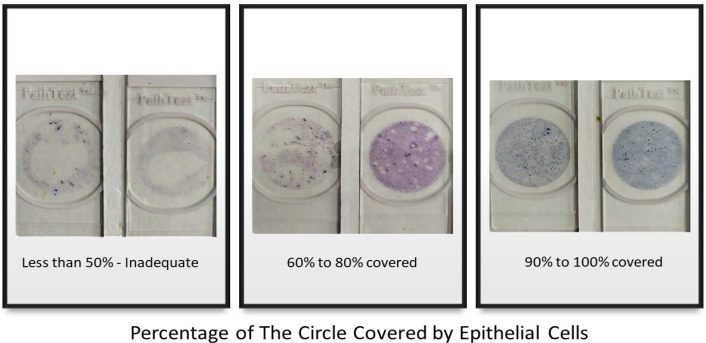
Images of Slides Showing Percentage of the Circle Covered by Epithelial Cells

A total of 400 patients were recruited in this study. The patients’ age range was between 19–77 years. The patients’ mean age was 43.4+/- and median age was 43. The most common age group for Pap smear screening was the age group of 40–49 years. Three hundred and eighty-one (95.25%) samples were considered adequate for interpretation. Nineteen (4.47%) samples were inadequate for interpretation and showed cell coverage of less than 50%. Two hundred and fifteen (53.75%) samples showed a 60%-80% percentage of cell coverage and 165 (41.25%) samples had 90%-100% cell coverage (Table 1 and Figure 1). Three hundred and sixty-eight (92%) samples showed good cellular distribution. The squamous epithelial cells appeared in single forms as well as in syncytial arrangements. Only 32 (8%) samples showed an uneven cell distribution; cells were not evenly scattered, with squamous cells showing crowding and overlapping in certain areas (Table 1). No samples had poor cellular distribution where the cells were unevenly scattered, crowded, and overlapped in most areas.

Two (0.5%) samples had inflammatory cells, which obscured more than 75% of the epithelial cells. Forty-four (11%) samples had inflammatory cells partially obscuring (50-70%) the squamous cells, and 354 (88.5%) samples had inflammatory cells without obscuring the squamous cells. Three hundred and eighty-three (95.75%) of the smears showed that erythrocytes were absent from the smear or only present in a small amount, which did not obscure the squamous cells at all.

Three hundred and eleven (77.75%) of all smears were negative for intraepithelial lesion (NILM) and 57 (14.25%) smears were reported to have been infected by either bacterial vaginosis, Actinomyces sp., Candida spp., or Trichomonas vaginalis. Thirteen (3.25%) cases were reported as abnormal smears, which varied from ASC-US (2 cases), ASC-H (1 case), LSIL (1 case), HSIL (1 case), AGC endocervical (4 cases), AGC endometrial (1 case), AGC NOS (1 case), AIS (1 case), to adenocarcinoma of endometrial (1 case). Among all cases, 123 cases (30.8%) had subsequent histopathology followed by 89 cases (72.4%) with concordant results. The data agreement was then analysed using chi-square test. 

Overall, almost all (99%) of the samples showed good performance ratings, scoring from 16 to 20. Only 4 (1%) samples had an average performance rating with a total score of 10 to 15. According to results of chi-square test, the relationship between total score and criteria for scoring and was significant (p<0.05). As it is clear from Table 2, we can conclude that the performance of samples was influenced by sample adequacy, percentage of the circles covered by epithelial cells, cellular distribution, obscuring factor-inflammatory cells, and obscuring factor-red blood cells.

**Figure 2:**
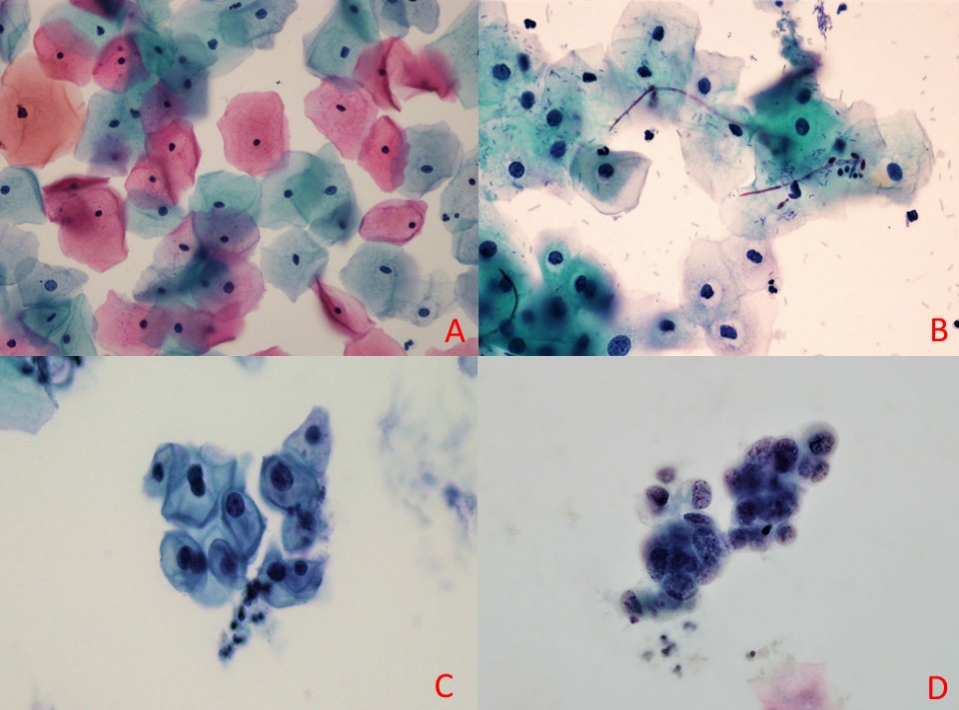
Images of Cells Taken from the Study. A, Normal cells under x40 magnification; B, Candida spp. infection at x40 magnification; C, Low grade squamous intraepithelial lesion [LSIL] cells at x40 magnification; D, Adenocarcinoma of endometrial cells at x40 magnification

**Table 1:** Evaluation Score and Percentage

Criteria	Categories	Number of cases (percentage)
i) Sample Adequacy	Inadequate cellularity	19 (4.75%)
	Adequate cellularity	381 (95.25%)
ii) Percentage of The Circle Covered by Epithelial Cells	Below 50% coverage	20 (5.00%)
	60% to 80% coverage	215 (53.75%)
	90% to 100% coverage	165 (41.25%)
iii) Cellular Distribution	Poor	0 (0%)
	Average	32 (8.00)
	Good	368 (92.00)
iv) Obscuring Factors	Presence of inflammatory cells.	
	Poor	2 (2.00)
	Average	44 (11.00)
	Good	354 (88.50)
	Presence of red blood cells (RBCs).	
	Poor	4 (1.00)
	Average	13 (3.25)
	Good	383 (95.75)
v) Cell Fixation	Nuclear features	
	Poor	0 (0)
	Good	400 (100)
	Cytoplasmic features	
	Poor	0 (0)
	Good	400 (100)
	Organisms (Bacteria, fungal and protozoa).	
	Poor	0 (0)
	Good	400 (100)

**Table 2:** The Relationship between Total Score and Criteria for Scoring

Variables	Total score			Crude or (95% CI)	P value, <
	Poor/ Inadequate	Average/ Adequate	Good		
Sample adequacy	19	381	N/A	0.936, 1.129	0.000
Percentage of The Circle Covered by Epithelial Cells	1	215	165	0.963, 1.033	0.000
Cellular Distribution	0	32	368	0.896,1.024	0.000
Obscuring factors: Inflammation	2	45	353	0.990,1.094	0.000
Obscuring factors: Blood	4	12	384	0.840,0.979	0.000
Age	-	-	-	-	0.841
HPE report concordant	-	-	-	-	0.969

## Discussion

Cervical cancer is one of the most fatal but completely preventable cancers in women. The Global Cancer Observatory (GLOBOCAN, 2020) has revealed that the incidence and mortality rate of cervical uteri cancer are 13.3% and 7.3%, respectively (Global Cancer Observatory, 2021). According to National Cancer Registry, the risk of developing cancer increases in women by aging. Women aged between 70-74 years old have the highest incidence rate of getting cervix uteri cancer (Ab Manan, et al. 2019). One of the most popular and effective methods for preventing cervical cancer is to have a regular cervical smear cytology (Pap Smear) check up every year or every three years after 2 consecutive negative smears. Women who are not regularly screened are 3 to 10 times more likely to get cervical cancer compared to women who are regularly screened (Mohd Taib et al., 2020). According to a previous study, invasive cervical cancer is rare in women under the age of 25 years old and cytological abnormalities in the cervix are common in younger women, which makes screening less cost-effective in women under 25 (Sasieni et al., 2003). Although vaccination for the HPV has been introduced in most countries, some countries still stick to pap smears as a preventive action due to financial constraints.

LBP slides have been proven to be superior to conventional smears because of their ability to remove excessive red blood cells, mucus, and inflammatory exudate from samples whilst maintaining cellular appearance. Moreover, the LBP smear produced a homogenous and uniform thin layer smear, which makes it easier for screening (Desai, 2014). Some researchers have reported that, despite the greater cost, liquid-based cytological preparation resulted in (a) a cleaner background smear, (b) good cell distribution, (c) well-preserved cytomorphology, (d) reduced screening time, (e) well-preserved cells in solution for longer storage time, and (f) decreased air-dry artifacts better than direct smear preparation (Nandini et al., 2012; Bibbo et al., 2015). Among all the LBP systems that exist nowadays, ThinPrep and SurePath remain as the most sought-after in the cytology community. There is no doubt that both systems, which have been established for decades, are almost perfect and offer better technology in terms of the imaging system and molecular testing. However, for a small laboratory with a tight budget, finding an alternative LBP system is a must. Thus, PathTezt was introduced as an alternative product that follows the principle of the ThinPrep system. The system offers an affordable price for the user and produces a good quality slide.

Based on the Bethesda System for Reporting Cervical Cytology 2014 (TBS 2014), specimen adequacy criteria is that liquid-based cytology smears should have more than 5,000 squamous or squamous metaplastic cells for the evaluation. Women who undergo chemotherapy or radiotherapy as well as those with post-menopausal having atrophic changes or post-hysterectomy may have samples with fewer than 5000 cells. Such specimens as previously mentioned should be considered as adequate at the discretion of the laboratory (Nayar, et al., 2015). We had quite a few unsatisfactory smears in this study. The reason for the unsatisfactory smear from this study was mainly due to scanty squamous cell components and thick inflammatory cells. Usage of lubricant and not removing blood before sampling are believed to be the reasons why the smears have a patchy or halo surface. The percentage of circles covered by epithelial cells is commensurate with the adequacy of the samples because when the percentage is lower, the fewer cells adhere to the slide.

The cellular distribution of the smears from this study also showed an admirable result. It was found that only 8% of the smears had squamous cells crowding and overlapping in certain areas, and no smear having cells that were not evenly scattered, crowded, and overlapped in most areas, revealing that the smears had high quality in terms of cellular distribution when compared to conventional smears. The dispersion and vacuum techniques are the main principles of the PathTezt system. These techniques are therefore effective and play an important role in minimizing obscuring factors, such as mucus, inflammatory cells, and red blood cells. The presence of inflammatory cells can be a clue to an existing infection or to unknown causes. There were only a few smears that had inflammatory cells and red blood cells that covered more than 75% of the smear in the background. This finding showed that almost all erythrocytes wer lysed during the processing step. These are the main features that make LBP cytology superior to conventional smears.

The PathTezt preservative solution is effective in preventing suboptimal fixation problems. PathTezt uses a methanol-based solution which provides a good preservation for the cells and reacts excellently with Papanicolaou’s staining solution. The nuclear details are crisp and cytoplasmic features are easily seen. Atypical cells and infective microorganisms, such as fungal elements (Candida sp.), bacteria (coccobacilli and colonies of Actinomyces sp.), and protozoa (Trichomonas vaginalis) are easily detected under microscopic examination. Figure 2 shows selected images taken from the slides in this study. Nuclear details and cytoplasmic differentiation could be appreciated easily.

Sample adequacy, percentage of cells covered by the smear, cellular distribution, and obscuring factors-inflammatory cells, and obscuring factors-red blood cells are the determining factors for a good quality smear. Good quality smears will help cytotechnologists and cytopathologists make a precise diagnosis, subsequently contributes to better patient management. This fact is evidenced by the cytologic-histologic correlation analysis that was done, which shows a significant p-value of <0.005.

*Limitations of the study*

We used a split-sample approach in this study, but the lack of an easy method to accurately divide the sample made this type of study impractical. We also have a limited fund to run the study. Thus, only a systemic evaluation seems feasible. 

In conclusion, cervical smear evaluation systems for cervical cancer screening can be challenging due to many factors. According to the findings of this study, the modified-LBC preparation could be an alternative laboratory method when commercial preparations are unaffordable. We also found that PathTezt LBP for cervical cytology was cost-effective, comparable, and acceptable in terms of quality of smears with the other first-generation LBP methods.

## Author Contribution Statement

Nur Syuhada: ran the labwork, collected data and analyzed data. Anani Aila: designed study, edited final version and reviewed the paper. All authors read an approved the final version. 
